# Supplementary material for: Prevalence and risk factors for human leptospirosis at a hospital serving a pastoralist community, Endulen, Tanzania
Source: PLoS Negl Trop Dis. 2023 Dec 20;17(12):e0011855. doi: 10.1371/journal.pntd.0011855 (PMC10766184; doi:10.1371/journal.pntd.0011855)
Supplement: S2 File — (DOCX) [file pntd.0011855.s002.docx]

**Supplementary File 2a. Correlation matrix of livestock related activities undertaken during the preceding month among patients presenting to Endulen Hospital with fever, 2016-17**

|  | Birthed cattle | Birthed goat | Birthed sheep | Milked cattle | Milked goats | Milked sheep | Slaughtered cattle | Slaughtered goats | Slaughtered sheep | Cleaned cattle waste | Cleaned goat waste | Cleaned sheep waste |
| --- | --- | --- | --- | --- | --- | --- | --- | --- | --- | --- | --- | --- |
| Birthed cattle | 1.00 |  |  |  |  |  |  |  |  |  |  |  |
| Birthed goat | 0.55 | 1.00 |  |  |  |  |  |  |  |  |  |  |
| Birthed sheep | 0.56 | 0.61 | 1.00 |  |  |  |  |  |  |  |  |  |
| Milked cattle | 0.16 | 0.09 | 0.01 | 1.00 |  |  |  |  |  |  |  |  |
| Milked goats | 0.12 | 0.21 | 0.21 | 0.41 | 1.00 |  |  |  |  |  |  |  |
| Milked sheep | 0.18 | 0.21 | 0.21 | 0.17 | 0.62 | 1.00 |  |  |  |  |  |  |
| Slaughtered cattle | 0.06 | 0.11 | -0.01 | 0.13 | 0.10 | -0.02 | 1.00 |  |  |  |  |  |
| Slaughtered goats | 0.26 | 0.27 | 0.23 | 0.18 | 0.23 | 0.15 | 0.53 | 1.00 |  |  |  |  |
| Slaughtered sheep | 0.23 | 0.27 | 0.22 | 0.27 | 0.34 | 0.25 | 0.38 | 0.69 | 1.00 |  |  |  |
| Cleaned cattle waste | 0.08 | 0.06 | 0.01 | 0.47 | 0.27 | 0.15 | 0.13 | 0.14 | 0.30 | 1.00 |  |  |
| Cleaned goat waste | 0.13 | 0.23 | 0.23 | 0.28 | 0.32 | 0.15 | 0.05 | 0.15 | 0.28 | 0.45 | 1.00 |  |
| Cleaned sheep waste | 0.10 | 0.24 | 0.19 | 0.32 | 0.30 | 0.12 | 0.08 | 0.17 | 0.33 | 0.55 | 0.91 | 1.00 |

**Supplementary File 2b. Correlation matrix of livestock related activities undertaken during the preceding year among patients presenting to Endulen Hospital with fever, 2016-17**

|  | Birthed cattle | Birthed goat | Birthed sheep | Milked cattle | Milked goats | Milked sheep | Slaughtered cattle | Slaughtered goats | Slaughtered sheep | Cleaned cattle waste | Cleaned goat waste | Cleaned sheep waste |
| --- | --- | --- | --- | --- | --- | --- | --- | --- | --- | --- | --- | --- |
| Birthed cattle | 1.00 |  |  |  |  |  |  |  |  |  |  |  |
| Birthed goat | 0.55 | 1.00 |  |  |  |  |  |  |  |  |  |  |
| Birthed sheep | 0.57 | 0.87 | 1.00 |  |  |  |  |  |  |  |  |  |
| Milked cattle | 0.22 | 0.25 | 0.21 | 1.00 |  |  |  |  |  |  |  |  |
| Milked goats | 0.07 | 0.10 | 0.13 | 0.64 | 1.00 |  |  |  |  |  |  |  |
| Milked sheep | 0.03 | 0.09 | 0.04 | 0.35 | 0.58 | 1.00 |  |  |  |  |  |  |
| Slaughtered cattle | 0.12 | 0.06 | 0.05 | 0.22 | 0.23 | 0.14 | 1.00 |  |  |  |  |  |
| Slaughtered goats | 0.16 | 0.14 | 0.11 | 0.23 | 0.19 | 0.13 | 0.81 | 1.00 |  |  |  |  |
| Slaughtered sheep | 0.11 | 0.10 | 0.05 | 0.24 | 0.24 | 0.20 | 0.73 | 0.81 | 1.00 |  |  |  |
| Cleaned cattle waste | 0.05 | -0.03 | 0.00 | 0.32 | 0.29 | 0.08 | 0.19 | 0.21 | 0.17 | 1.00 |  |  |
| Cleaned goat waste | 0.13 | 0.20 | 0.19 | 0.36 | 0.34 | 0.09 | 0.08 | 0.14 | 0.13 | 0.54 | 1.00 |  |
| Cleaned sheep waste | 0.11 | 0.18 | 0.17 | 0.39 | 0.39 | 0.07 | 0.07 | 0.13 | 0.13 | 0.60 | 0.94 | 1.00 |
