# Supplementary material for: Prevalence and risk factors for human leptospirosis at a hospital serving a pastoralist community, Endulen, Tanzania
Source: PLoS Negl Trop Dis. 2023 Dec 20;17(12):e0011855. doi: 10.1371/journal.pntd.0011855 (PMC10766184; doi:10.1371/journal.pntd.0011855)
Supplement: Attachment — Submitted filename: Endulenlepto_coverletter_27Oct23.docx [file pntd.0011855.s003.docx]

Drs Kamhawi and Brindley

Editors-in-Chief

PLoS Neglected Tropical Diseases

28 October 2023

**Re: PNTD-D-23-01043: Prevalence and risk factors for human leptospirosis at a hospital serving a pastoralist community, Endulen, Tanzania**

Dear Editors,

Thank you for the opportunity to provide a revised manuscript. We have carefully considered the reviewers comments and made a number of changes to the manuscript. Each of the reviewers’ comments is addressed below, with changes in the manuscript highlighted.

This report has not been previously published, nor is the manuscript under consideration, nor been accepted for publication elsewhere. All authors have contributed substantially to the work, fulfil the criteria for authorship, have seen and approved the version of the manuscript enclosed and concur with its submission. I am the corresponding author and had full access to all the data in the study and had final responsibility for the decision to submit for publication.

Yours sincerely

Michael Maze, MB ChB, DCH, DTM&H, PhD, FRACP

Department of Medicine and Centre for International Health

University of Otago

New Zealand

**Reviewer #1:**

**Comment:** The objectives of the study are clearly articulated. No hypothesis was tested in this study. The study population is well-stated; however, it may not align entirely with the study's objectives. Specifically, the study population comprises patients who visited Endulen Hospital, and this might not accurately represent the broader pastoral community within the Ngorongoro Conservation Area (NCA). Consequently, this approach could introduce bias into the prevalence estimates and effect sizes, as patients with symptoms and probably Leptospira seropositive are more inclined to seek medical care at the hospital. To enhance clarity, I recommend a more precise articulation of the study population, particularly in the study objectives and title. It's essential to explicitly state that the study is hospital-based rather than community-based. Moreover, a community-based study utilizing random sampling techniques would have been better suited to achieve the study's objectives and provide a more representative understanding of the NCA pastoral community's leptospirosis dynamics.

**Response:** We have adjusted the study title to explicitly state that the study is hospital based. It now reads: “Prevalence and risk factors for human leptospirosis at a hospital serving a pastoralist community, Endulen, Tanzania”.

We have also adjusted the manuscript to acknowledge the sampling limitations of hospital based studies in the manuscript (Line 262-264): “We used hospital based surveillance in order to detect acute leptospirosis. A limitation of this approach is that seroprevalence of the study population may differ from the community and care must be taken if comparing our seroprevalence estimate to population-based serosurveys.”

**Comment:** The absence of information regarding the necessary sample size is noteworthy. I strongly advise the authors to provide justification for the enrollment of the specified number of participants. It's important to clarify whether there was a systematic procedure used for sample size estimation. If the study relied on convenience sampling, I recommend that the authors explicitly acknowledge the limitations on their study findings associated with this sampling method within the limitations section. This will help readers understand the potential impact of the sampling approach on the study's outcomes.

**Response:** Leptospirosis analyses were a secondary objective of the main study, which was originally powered to estimate the prevalence of brucellosis in this population. The original sample size calculations were as follows: “Based on an assumed prevalence of 9%, it was estimated that a sample of 126 individuals would be required to estimate brucellosis case prevalence with precision of 5% and 80% power.” Given plausibly similar prevalence of leptospirosis as brucellosis in this population (e.g. 10.1% of adults and adolescents enrolled in a hospital based study in Moshi 2007-8. Crump et al 2013, PLoS Negl Trop Dis) we are confident that the sample available for this study is sufficient to meet the primary aim of prevalence estimation. We have modified the manuscript to highlight this limitation (line 262-272): “Further, leptospirosis testing and analysis was a secondary aim of the study and the study was not subject to *a priori* sample size calculations for leptospirosis prevalecne estiamation or risk factor identification specifically. The foundation study was powered to investigated the epidemiology of brucellosis in this hospital population. One component of the original study was powered with required sample size of 126 to estimate prevalence, assuming a prevalence of 9%, desired precision of 5% and power of 80% [24]. Given plausibly similar expected prevalence of brucellosis and leptospirosis in this population [34] and availability of data from of 229 participants it is unlikely that this study was underpowered for the prevalence estimation goal but it may be underpowered to identify some of the associations assessed in the risk factor analysis.”

**Comment:** The results are completely reported. However, In lines 169-171 the authors mention about evaluating Interactions between history of livestock death and livestock behavior variables for both outcomes but they never refer to this anywhere in the results section. If this analysis was conducted, i suggested the results are reported.

**Response:** We agree and have added the results of our evaluation of interactions to the manuscript in the relative location for the two analyses: (line 216 and line 219-220 ): “No significant interaction terms were identified.”

**Comment:**  The conclusions are supported by the data. Limitation were described. However, I suggest the authors, highlight in their limitations the drawbacks of relying on hospital visits to estimate the seroprevalence, given the asymptomatic nature of leptospirosis infection in most of the infected cases.

**Response:** We agree that estimates of seroprevalence are potentially biased, and have added this to the discussion (lines 262-64): “We used hospital based surveillance in order to detect acute leptospirosis. A limitation of this approach is that seroprevalence of the study population may differ from the community and care must be taken if comparing our seroprevalence estimate to population-based serosurveys.”

**Comment:** Overall, this study holds significant importance as it addresses a critical gap in leptospirosis-related research within sub-Saharan Africa. This region has seen a substantial underestimation of the infection's prevalence, both in humans and livestock. Notably, the authors shed light on the pivotal role played by livestock in the transmission dynamics of leptospirosis. Further illustrated by the directed acyclic graph presented in the supplementary figure. The DAG is quite informative, if possible this could be included as a figure in the main text and not among supplementary. The study underscores the pressing need for further studies and interventions, employing a one-health approach to tackle this complex issue.

Nonetheless, I have proposed some revisions in the subsections above, which I encourage the authors to carefully consider.

**Response:** Thank you for the considered suggestions. The response to your comments and proposed revisions are detailed above. In response to your request we have moved the DAG to the main manuscript.

**Reviewer #2:**

**Comment:** The objectives and the design was appropriate for the study. However, some issues with methodology, especially study population lacks details and makes it difficult to appreciate the rural aspect mentioned in this paper and community/household set up. The geographic map provided shows the location of the Endulin Hospital and lacks important geographical features important in understanding the disease dynamics. Enrolled patients/ participants were followed up to their homes/communities but no information on the location in relation to the hospital. A better map is needed to show patients location in relation to hospital and other geographic features like water sources, roads, forests etc

**Response:** We have included a smaller scaled map to better appreciate the geography of the region. Geospatial analyses are beyond the scope of this study, but we agree may add to understanding.

**Comment:** I have concerns on eligibility criteria where they enrolled from 2 years. Several issues arise of ethical and appropriateness based on the known lepto epidemiology and risks of exposure.

**Response:** We agree that potential exposures among children are likely to differ from adults, but we considered children an important group for investigation due to strong local evidence that leptospirosis is a cause of severe febrile illness among children. For example, in Kilimanjaro Region where leptospirosis incidence has been estimated as high as 175 cases per 100,000 children aged <5 years (Biggs 2013), and in a connected study, among confirmed paediatric cases of leptospirosis the median age was 2.4 years (Biggs et al 2011).

**Comment:** This raises the issues of assent and consent especially for young ages from 2 year to 18 years which require different levels of asset and consent with the involvement of the guardians. It is further mentioned that the consent was written. Were all participants able to read, understand and consent?

**Response:** All participants, or their legal guardians provided informed written consent following a rigorous consent process. Our study underwent review, and was approved, by ethics committees at Tanzanian institutions involved in the study and at the National Institute for Medical Research. Consent forms were available in Kiswahili (the national language of Tanzania). In case of participants being unable to read or understand Kiswahili, study staff were also fluent in Maa (the first language of Maasai) and were able to translate. In the case of children, consent was obtained from their guardians, and where appropriate, assent was also gained from the children. To ensure that this is clear to readers we have added the following content on lines 191-194 “Written informed consent was obtained from all adult participants, with forms translated into Kiswahili and verbal Maa translation also provided as needed. Informed consent for minors (under 18 years old) was provided by their parent or guardian and assent was additionally obtained from all participants aged 13-17 years old.”

**Comment:** Secondly, it is mentioned that the questionnaire was administered. Which language was used for the questionnaire? |Evidence of the interpreted questionnaire in local languages should be provided.

**Response:** The questionnaire was administered by trained study staff in either Kiswahili or Maa. The written version of the questionnaire was read to participants by study staff, rather than directly by participants. The form, in both English and Kiswahili is attached as Supplementary File 1.

**Comment:** Details on sample collection, transportation and preservation before shipment is lacking?. Samples were shipped to USA for analysis? why. Further, evidence of material transfer agreement need to be provided.

**Response:** Details of sample collection, preservation and transport are in the manuscript: (line 126-128) “Enrolled patients underwent phlebotomy, following skin sterilisation with isopropyl alcohol and povidine iodine. Blood samples were prioritised for culture and malaria testing. A target volume of 10 mL blood was then placed into a plain vacutainer (BD, Franklin Lakes, NJ, USA) for serology.” ; (line 139-140) “Sera were stored at -80°C and shipped on dry ice prior to serological testing at the US Centers for Disease Control and Prevention (Atlanta, GA, United States).”

We acknowledge and fully support the importance of building local laboratory capacity and have been involved in development of multiple assays at the Kilimanjaro Clinical Research Institute. On this occasion, however, samples were shipped to the US CDC as an international reference centre for testing due to longstanding collaborative links. A Material Transfer Agreement (NCEZID-136933-00) has been signed by all relevant parties. The manuscript has been revised to include this information (lines 194-196): “Sample shipments between Tanzania and the United States were performed in accordance with the Material Transfer Agreement between Kilimanjaro Christian Medical Centre and the US Centers for Disease Control and Prevention (NCEZID-136933-00).”

**Comment:** Patients aged ≥2 years were eligible to enrol if they had a history of fever within 72 hours, or a measured tympanic temperature of ≥38.0°C at presentation. Fever may be caused by several infections and in Africa settings mainly by Malaria. Therefore the study needed to ensure that enrolled patients with febrile illness were malaria negative as part of the criteria especially since these were outpatients.

**Response:** We agree that malaria is an important diagnosis to exclude in sub-Saharan Africa. Malaria testing was performed using SD BIOLINE Malaria Ag P.f/Pan rapid diagnostic test (Standard Diagnostics/Abbott, Abbott Park, IL, USA) or CareStart Malaria HRP2 (Pf) (ACCESS BIO, INC. Somerset, NJ, USA). These results are previously reported (Bodenham et al 2020 – reference 24 in the revised manuscript). Notably the prevalence of positive malaria testing was lower than leptospirosis (2.6% vs 7.9%).

We have modified the manuscript to clarify that malaria testing was performed (lines 136-139): “Malaria rapid diagnostic testing was performed directly from the sample collection syringe using the SD BIOLINE Malaria Ag Pf/Pan rapid diagnostic test (Standard Diagnostics/Abbott, Abbott Park, IL, USA) or CareStart Malaria HRP2 (Pf) (ACCESS BIO, INC. Somerset, NJ, USA). Blood culture and malaria testing results have been previously presented [24].” We have also added data to indicate that none of the six participants with a positive malaria antigen test met our case definition of leptospirosis (Table 1).

**Comment:** Whereas we know and appreciate the leptospirosis is one of the causes of the febrile illness, a lot is still needed to understand the role of the different serovars and serogroups in the casuation of the disease and the clinical presentation. This conclusion "Leptospirosis was a common cause of febrile illness in this rural community. Interventions that reduce risks associated with milking livestock may reduce human infections" is overstated and should be revised. The same applies to the open statement in the abstract. What is the policy recommendation from this study given that your study reports "18 (7.9%) cases of acute leptospirosis, with Djasiman 8 (44.4%) and Australis 7 (38.9%) the most common predominant reactive serogrups".

**Response:** We have revised the abstract conclusion to read “We identified leptospirosis in approximately one in twelve patients attending hospital with fever from this rural community. Interventions that reduce risks associated with milking livestock may reduce human infections.” And altered the relevant section of the Author Summary to read “We found 8% had leptospirosis and nearly 1 in 3 people had been previously exposed. Milking cattle and goats were risk factors for leptospirosis or previous exposure to leptospirosis We conclude that that livestock are a likely source of human infection and that milking livestock is a risk factor for being infected”

**Comment:** Your study followed patients to collect samples in their households, now 18 patients were found to have acute leptospirosis based on the titers. No other clinical symptoms are described. what is our ethical obligations?

**Response:** Our study was primarily hospital based and all patients were assessed and treated by trained clinicians during their illness. The results of study tests including blood culture findings and malaria antigen tests were made available to treating clinicians during the illness with guidance on the interpretation of tests (and appropriate management options for identified pathogens) that were not routinely in use at this hospital. Leptospirosis testing was conducted several months after the acute illness. As these would not influence patient management at the time they were tested, and in keeping with the ethics committee recommendations and participant consent, these results were not returned to participants. We agree that access is needed to reliable leptospirosis tests with a clinically meaningful turn-around-time.

**Comment:** The study is very important and adds more information to the previously limited published studies. This study combines hospital and patient follow up in the community which is a very good design. However, the authors could have collected more data on the enrolled patients including the symptoms, rule out malaria and probably could have obtained the clinical picture of the patients. Now their conclusions is based on cutoff titres defined in this study and as previously reported only which makes the conclusion weak especially given different studies are reporting different serovars. This study specifically reports Djasiman as being responsible the acute leptospirosis and is not well described in Africa and later on appreciating its role in leading to acute disease.

**Response:** Thank you for the positive comments. With regards to collecting information on symptoms, we highlight Table 1, where patient symptoms are detailed and where we have now included information on malaria testing. With regards to MAT serology, we acknowledge your comment about the cut-off titre and specifically highlight in the manuscript, the advantages and disadvantages of our use of a titre of ≥400 as a diagnostic cut-off. We highlight that this cut-off is the World Health Organisation standard and widely used in clinical and research practice. MAT serology is generally considered serogroup specific and we consider the serogroup findings to be robust. However, we acknowledge that without isolates to confirm the presence of specific serovars conclusions need to be made cautiously.

**Reviewer #3:**

**Comment:** The keywords in the system and the manuscript were not match.

**Response:** We have adjusted the manuscript key words to match the system.

**Comment:** Some patient information is missing. Do some patients have underlying diseases? Smoking? Or antibiotic use?

**Response:** We have added prior antimicrobial medication use to Table 1. The prevalence of co-morbidities and smoking status was not collected.

**Comment:** The detection method is dependent on MAT, which makes the research conclusion less reliable. In the diagnosis of acute leptospirosis, it is better to combine molecular diagnostic techniques.

**Response:** Micro-agglutination testing is a reference standard diagnostic test for leptospirosis and we consider the results reliable. We agree that the addition of molecular testing would have further increased the sensitivity of our diagnostic testing and potentially increased the estimated disease prevalence. In our study discussion we highlight this limitation: “Despite previous studies in Tanzania finding *Leptospira* nucleic acid amplification testing (NAAT) of blood and urine of infected patients had low sensitivity of to diagnose leptospirosis [11], NAAT may be a useful addition to MAT serology in future studies estimating leptospirosis prevalence [36].”

**Comment:** Although the serums were tested at the US Centers for Disease Control and Prevention, it is suggested to write the method of MAT clearly.

**Response:** We have added further information to describe details of the MAT serology method.The manuscript now reads: (lines 140-154) “Serology for anti-Leptospira antibodies was performed on acute and convalescent serum samples using the standard microscopic agglutination test (MAT) [25]. Serial dilutions of participant serum were made in microtitre plates beginning at a dilution of 1:100, with subsequent two-fold dilutions. An equal volume of live leptospires were added, with a panel of 20 *Leptospira* serovars belonging to 17 serogroups. The MAT panel included serogroups: Australis (represented by *L. interrogans* serovar Australis, *L. interrogans* serovar Bratislava), Autumnalis (*L. interrogans* serovar Autumnalis), Ballum (*L. borgpetersenii* serovar Ballum), Bataviae (*L. interrogans* serovar Bataviae), Canicola (*L. interrogans* serovar Canicola), Celledoni (*L. weilii* serovar Celledoni), Cynopteri (*L. kirschneri* serovar Cynopteri), Djasiman (*L. interrogans* serovar Djasiman), Grippotyphosa (*L. interrogans* serovar Grippotyphosa), Hebdomadis (*L. santarosai* serovar Borincana), Icterohaemorrhagiae (*L. interrogans* serovar Mankarso, *L. interrogans* serovar Icterohaemorrhagiae), Javanica (*L. borgpetersenii* serovar Javanica), Mini (*L. santarosai* serovar Georgia), Pomona (*L. interrogans* serovar Pomona), Pyrogenes (*L. interrogans* serovar Pyrogenes, *L.* *santarosai* serovar Alexi), Sejroe (*L. interrogans* serovar Wolffi), and Tarassovi (*L. borgpetersenii* serovar Tarassovi). Plates were read microscopically by estimating 50% agglutination as the end-point titre. Negative controls and positive controls were performed with every run.”

**Comment:** In the methods section, it was written that patients aged >2 years were eligible. However, in the results section, it was written the median (range) age was 27 (0,78) years.

**Response:** Thank you for identifying errors in our age data. We have reviewed the source documents and identified that the participant documented as 0 years should have been recorded as 9 years. This has been corrected in the database and the manuscript (Table 1).

**Comment:** Another question to consider is, are young children more likely to become infected from milking on the farm?

**Response:** We agree that the transmission pathways of leptospirosis may differ by age group. In our study very few young children participated in milking, and very few met our case definition for leptospirosis.

**Comment:** An interesting phenomenon is that the serogroups of acute leptospirosis and seropositivity to Leptospira serovars were different. The authors should further discuss this discrepancy which may be the potential causes, to make better strategies for prevention and control of leptospirosis.

**Response:** We agree that this is an interesting phenomenon, and discuss possible reasons for this result in our discussion (lines 243-45): “The discrepancy between acute cases and seropositivity may indicate that a *Leptospira* serovar from Icterohaemorrhagiae serogroup was previously a more common cause of acute disease, and is in keeping with other evidence suggesting that the relative importance of serovars may change over time [6].”

**Comment:** Since the prevalence of leptospirosis in livestock is also unclear, it is difficult to conclude that human leptospirosis is related to the transmission of livestock.

**Response:** We reach the conclusion that livestock is a probable source of human infection based on our risk factor analysis. We agree that further data is needed, particularly data that serologically or genomically links organisms detected in humans and livestock. In response to your concern we have added the following comment to our discussion (lines 260-261): “Similar work to link *Leptospira* from animals and humans is important step for future research in our study area.”

**Reviewer #4:**

**Comment:** In the abstract, the potential association between infection with leptospirosis and pastoralist animal husbandry practices and sustained livestock contact is not clearly explained, so the proposal for the purpose of the study appears abrupt.

**Response:** Although the abstract word count precludes extensive explanation, we have expanded the rationale to more clearly explain the study purpose. It now reads (lines 40-43): “Leptospirosis is suspected to be a major cause of illness in rural Tanzania associated with close contact with livestock. We sought to determine leptospirosis prevalence, identify infecting Leptospira serogroups, and investigate risk factors for leptospirosis in a rural area of Tanzania where pastoralist animal husbandry practices and sustained livestock contact are common.”

**Comment:** The study design was appropriate to address the stated objectives. The population wasn't clearly described for the hypothesis being tested. The sample size was sufficient and the statistical analysis was correct. There were no concern about ethical or regulatory requirements being met.

**Response:** Thank you for identifying potentially inadequate description of the population. We have adjusted the study title to clarify the hospital based nature of the study. We have further described the population in the methods section with regards to eligibility criteria (lines 121-124): “Patients seeking care at the outpatient department of Endulen Hospital from August 2016 through October 2017 were eligible for screening. Screening was performed on 259 of 422 days in the screening period (4-5 days in each week). Patients aged ≥2 years were eligible to enrol if they had a history of fever within 72 hours, or a measured tympanic temperature of ≥38.0°C at presentation.” In the results section, we describe the population characteristics in Table 1. Further information on co-morbidities is not available.

**Comment:** The presented analysis just met the lower limit of the analysis plan, the results need to presented more clearly and completed. The figures (Tables, Images) were with sufficient clarity.

**Response:** The descriptive analyses and results of logistic regression are included in Tables 1 and 2. The assessment of correlations is included as supplementary tables. To more clearly and completely describe results we have now also included the results of assessments of interaction terms.

**Comment:** Page 4, Line 66, The sentence "Leptospirosis is a zoonotic disease and major cause of human illness in sub-Saharan Africa [1, 2]." is unclear. Please re-word.

**Response:** We have re-worded this sentence for clarity, separating the ideas to two paragraphs. The text now reads (line 70): “Leptospirosis is a major cause of human illness in sub-Saharan Africa [1, 2];” and (line 82) Leptospirosis is a zoonotic disease with a wide variety of animals potential sources of human infection.

**Comment: Page** 4-5, Line 91-99, The content of this paragraph is a bit verbose，the wording should be updated.

**Response:** We have revised the paragraph to reduce verbosity. It now reads “Humans are incidental hosts of *Leptospira* and can be infected through exposure to either infected animals or to a contaminated environment [20]. Knowledge of specific risk behaviours or activities is essential for interventions to reduce human disease. Although risk factors for leptospirosis are broadly established [21], and include diverse exposures to rodents, livestock, and floodwaters, the importance of specific risk factors varies substantially by context due to variation in animal reservoirs and human activities. In areas of Tanzania where small-holder farming predominates [22], acute leptospirosis has been associated with activities such as cleaning cattle waste and feeding cattle [10]. Exploration of livestock-associated risk factors among pastoralist communities may provide actionable public health interventions to reduce the incidence of leptospirosis among high risk communities.”

**Comment:** The reference format of this article is questionable and please check it well.

**Response:** We have rechecked the references to ensure they meet PLoS formatting requirements, and think they do so. If there are any that do not meet specification we will work with the PLoS editorial team to correct these following acceptance.

**Comment:** Please engage an English language service to correct the grammar.

**Response:** We have rechecked the grammar within the manuscript. As many of the authors have English as their first language, we do not consider a grammar service is required, but would be willing to consider any suggested adjustments.

**Comment:** The research content of this article has a positive effect on improving the perception of Tanzanian people about the risk of leptospirosis infection and protecting relevant practitioners to reduce the chance of leptospirosis, which has certain significance. However, there is still room for optimization in article writing, language organization, etc., and it is recommended to receive it after modification.

**Response:** Thank you. We have made a number of modifications to the manuscript as detailed above.
